# Associations between self-reported oral health and incident stroke: a prospective analysis of the UK Biobank

**DOI:** 10.1186/s12889-026-26397-2

**Published:** 2026-02-05

**Authors:** Chang-Qing Sun, Hui-Min Liu, Qian-Yu Zhou, Meng-Ting Liu, Jia-Jun Chen, Peng Wang, Hua Ye, Qiang Zhang

**Affiliations:** 1 High-Tech Development Zone of States, School of Nursing and Health, Zhengzhou University, NO. 101 Kexue Road, Zhengzhou City, 450001 People’s Republic of China; 2 Department of Neurology, The First Affiliated Hospital of Zhengzhou University, Zhengzhou, 450052 Henan China; 3 Department of Epidemiology and Biostatistics, College of Public Health, Zhengzhou UniversityHigh-Tech Development Zone of States, NO. 100 Kexue Road, Zhengzhou City, 450001 People’s Republic of China; 4 Human Resources Department, the First Affiliated Hospital of Zhengzhou University, NO.1 Longhu Middle Road, Jinshui District, Zhengzhou City, 450052 People’s Republic of China

**Keywords:** Oral Health, Stroke, Self-Report, Prospective Cohort Study

## Abstract

**Background and purpose:**

This study aims to explore whether different types of oral conditions are associated with incident stroke risk.

**Methods and results:**

This cohort study included 476,868 individuals without outcome events at baseline, multivariable models were constructed using Cox proportional hazard regression to assess the association between different oral conditions and the incidence of stroke, ischemic stroke (IS) and myocardial infarction (MI). Our fully adjusted model showed that individuals with painful gums [HR_painful gums_: 1.31 (1.06, 1.63)], loose teeth [HR_loose teeth_: 1.45 (1.20, 1.77)] and dentures [HR_dentures_: 1.23 (1.12, 1.36)] have increased risk for stroke incidence.

**Conclusions:**

Individuals with painful gums, loose teeth and dentures have increased risk for stroke, IS and MI incidence, this study helps to identify high-risk stroke participants.

**Supplementary Information:**

The online version contains supplementary material available at 10.1186/s12889-026-26397-2.

## Introduction

Oral health is integral to overall wellbeing, yet oral conditions affect over 3.5 billion people worldwide [1], posing a substantial global public health and economic burden [2]. Recent systematic reviews and large-scale cohort studies have substantiated the link between the spectrum of oral health conditions—ranging from inflammatory conditions like periodontitis to end-stage tooth loss—and increased risk of stroke [3–7]. Even composite measures of poor oral health, including transient symptoms, have been linked to stroke risk, suggesting a broader connection between the oral cavity environment and cerebrovascular health.

Beyond stroke, poor oral health is increasingly recognized as a risk factor for multiple chronic inflammation-driven diseases such as type 2 diabetes, inflammatory bowel disease, Alzheimer disease, cardiovascular diseases and cancer [8–11]. However, the specific association with stroke—particularly ischemic stroke, which constitutes approximately 80% of all cases—remains inconsistent. While prospective cohorts report modestly elevated risks with periodontal disease [12, 13], two-sample mendelian randomization (MR) analyses do not support causality [14–16]. These discrepancies may stem from heterogeneity in the definitions of oral exposures and residual confounding in observational studies.

Therefore, we conducted a prospective analysis using the UK Biobank—a large-scale cohort with robustly ascertained data, sufficient statistical power, and rich phenotyping (including lifestyle, comorbidities, and biomarkers). This resource enabled comprehensive confounder adjustment to minimize residual bias and strengthen causal inference, allowing us to rigorously examine the association between baseline oral conditions and incident stroke.

## Material and methods

### Study population and the outcomes

**Fig. 1:**
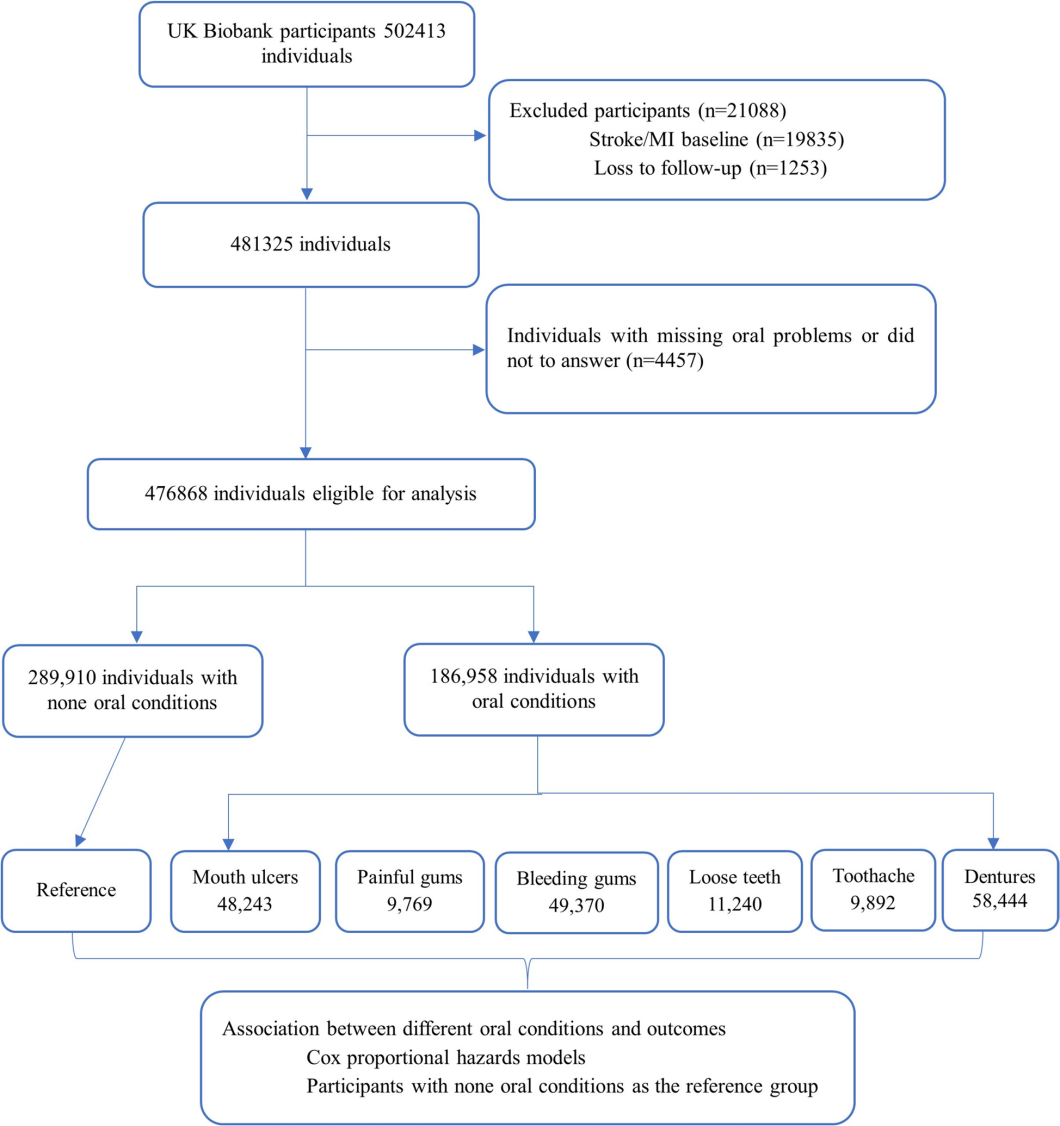
Flowchart of the participants selection

The UKB dataset is a large prospective cohort study which consists of more than 500,000 individuals aged 40–69 years with the baseline assessment between 2006 to 2010 [17]. The participants were recruited from 22 different assessment centers across England, Scotland and Wales in UK, through touch-screen questionnaire, participants provided their basic population characteristics, biological samples, genomics data and multiple health-related outcomes. In the current study, participants with stroke and myocardial infraction at baseline (occurrence date was before the date of first attendance to a UKB assessment center, n = 19,835) and those without follow-up information (n = 1253) and participants without oral conditions information were excluded (n = 4457). Finally, a total of 476,868 individuals were included for the analysis (Fig. 1).

This study included three outcomes, total stroke, myocardial infarction (MI), and IS. The primary outcome for this study is stroke incidence, which was extracted from “algorithmically defined outcomes” (Field ID 42006). The diagnosis of all outcomes was obtained through linkage with hospital inpatient records, death registers, primary care records, and self-reported. All outcome events were diagnosed according to their International Classification of Diseases 10th revision code (ICD code: stroke (I64), MI (I21), IS (I63)). Follow-up time was started from the date at first attendance to the assessment centre to the earliest date of stroke, MI, IS diagnosis, death or the censor date, whichever occurred first.

### Mouth problems assessment

The participants were asked about the question of “During the past year, do you have any of the following problems?”. The answers included “mouth ulcers, painful gums, bleeding gums, loose teeth, toothache and dentures” and additional options “none of the above and prefer not to answer”, those who prefer not to answer were excluded from this study. Therefore, according to whether with or without the mentioned oral conditions, participants were classified into seven groups: mouth ulcers, painful gums, bleeding gums, loose teeth, toothache, dentures (proxy for severe, irreversible tooth loss) and without oral conditions. To guide the biological interpretation of the associations, these exposure groups were considered under a conceptual framework of transient conditions (ulcers, toothache), periodontal indicators (painful gums, bleeding gums, loose teeth), and end-stage tooth loss (dentures).​

### Covariates

This study included three group of covariates. First group included basic demographic information, age, gender, ethnic background (British and non-British). Second group further included Townsend deprivation index (TDI is a socio-economic indicator used to measure the relative deprivation of individuals or regions in terms of resource access, primarily used to assess poverty levels and socio-economic inequality. The higher the score, the higher the degree of deprivation of an individual or region), employment status (employed, unemployed, and retired), average total household income (less than 18,000, 18,000 to 30,999, 31,000 to 51,999, 52,000 to 100,000 and greater than 100,000), smoking and alcohol drinking status (never, previous and current) and activity status measured by IPAQ questionnaire (low, moderate and high). Third group further included comorbidities (History of asthma, hay fever and eczema were also included as negative control exposures to help assess the specificity of the associations. A lack of strong association between these conditions and the outcome would increase confidence that a positive finding for oral conditions is not solely driven by unmeasured confounding or reporting bias) and medication status, diabetes (yes, no), cancer (yes, no), pain relief medication (yes, no), hypertension (≥ 140/90 mmHg, yes, no) and obesity (normal, underweight, overweight and obesity). All comorbidities were defined using data from the UK Biobank initial assessment visit (2006–2010), based on a combination of self-reported medical history, medication use, and touch-screen questionnaire data.

### Statistical analyses

The baseline characteristics of the participants were described using median and interquartile range (IQR) for continuous data, and category data was reported as frequency and percentage percentages. Association between different types of oral conditions and the outcomes (stroke, MI and IS) was estimated using Cox proportional hazards models and 95% CIs, participants who reported as none oral conditions were as the reference group. Schoenfeld residuals method was applied to test the assumption for the proportional hazards, for variables that might have been violated the PH assumption, a product term of time with that variable would be included into the model. Model 1 was adjusted for age, gender, ethic. Model 2 was additionally adjusted for Townsend deprivation index, employment status, average total household income, smoking and alcohol drinking status and activity status. Model 3 was further adjusted for hypertension, obesity, diabetes, cancer, and medication. We utilized complete-case analysis for multivariate Cox regression models. As a result, the sample size differs across three models. This is because participants with missing values in any of the covariates for a specific model were excluded from that model's analysis. The introduction of additional covariates in Models 2 and 3, particularly those related to lifestyle which had a proportion of missing data, led to a reduction in the analytical sample size for these models compared to Model 1. To account for multiple testing across the six primary oral condition exposures, which are interrelated, *P* values were adjusted using the Benjamini–Hochberg procedure to control the false discovery rate (FDR). The association between oral conditions and MI and IS was also assessed using the above final model.

However, to further validate the robustness of our findings despite the missing data, we performed a sensitivity analysis using multiple imputation with a random forest approach to account for the missing covariates. Additionally, since dentures represent a treatment modality rather than a direct oral health problem, we conducted a rigorous sensitivity analysis by completely excluding all participants reporting denture use.

To estimate the hypothetical population-level risk attributable to oral conditions, we calculated the population attributable risk percent (PAR%). The PAR% estimates the proportion of stroke cases that could theoretically be prevented if oral conditions were completely eliminated from the population, assuming a causal relationship. However, as this is an observational study, this calculation does not establish causality and should be interpreted as a theoretical maximum rather than a predictable effect of intervention. Despite this limitation, PAR% provides valuable insight for public health prioritization. The calculation was based on the fully adjusted HR from Model 3 and the prevalence of the exposure (${P}_{e}$) among the stroke cases in our cohort, using the following standard formula:$PAR\%=\frac{{P}_{e}(HR-1)}{{P}_{e}\left(HR-1\right)+1}$,Where ${P}_{e}$ is the prevalence of the exposure among the stroke cases in our study population, $HR$ is the fully adjusted hazard ratio for the association between the exposure and stroke. All the statistical analysis was performed in RStudio.

## Results

### Population characteristics

The participants selection procedure was illustrated in Fig. 1. Table 1 showed the baseline characteristic of the study participants according to different types of oral conditions. Among the 476,868 participants included in this study, 186,958 of them (39.21%) reported with oral conditions. The median follow-up period was 11.3 years (IQR: 1.4) for stroke incidence. Compared to those without oral conditions, those with different types of oral conditions are more likely to be female, unemployed, current smokers, have a higher Townsend deprivation index, BMI and education score. Participants with painful gums, loose teeth and dentures are more likely to be older, have lower income, have higher prevalence of diabetes, angina and high blood pressure. For participants with oral conditions, 4,415, 6959 and 3570 of them developed into stroke, MI and IS, respectively, the detailed characteristics by different outcomes events are shown in Supplementary Table 1, 2 and 3.

**Table 1:** Baseline characteristics of Study Patients by Different Oral Conditions

**Characteristic**	**Overall**, N = 476,868	**None of the above**, *N* = 289,910	**Mouth ulcers**, *N* = 48,243	**Painful gums**, *N* = 9,769	**Bleeding gums**, *N* = 49,370	**Loose teeth**, *N* = 11,240	**Toothache**, *N* = 9,892	**Dentures**, *N* = 58,444
Age (years)	56.35 (8.09)	55.76 (8.05)	55.65 (8.24)	55.98 (7.96)	54.26 (7.83)	58.24 (7.38)	54.56 (8.21)	61.63 (6.11)
Female: N (%)	264,268 (55%)	157,434 (54%)	28,946 (60%)	6,254 (64%)	30,790 (62%)	5,458 (49%)	4,928 (50%)	30,458 (52%)
British: N (%)	420,844 (88%)	257,835 (89%)	43,030 (89%)	7,799 (80%)	42,442 (86%)	9,083 (81%)	7,826 (79%)	52,829 (90%)
Townsend deprivation index	−1.33 (3.07)	−1.55 (2.95)	−1.34 (3.03)	−0.58 (3.39)	−1.07 (3.17)	−0.32 (3.47)	−0.80 (3.36)	−0.85 (3.27)
Education Score	15.36 (15.97)	14.08 (15.03)	15.20 (15.85)	18.23 (17.61)	16.23 (16.49)	19.74 (18.56)	16.70 (17.07)	19.75 (18.11)
Household income: N (%) ($)								
Less than 18,000	90,052 (19%)	45,487 (16%)	9,240 (19%)	2,506 (26%)	8,757 (18%)	3,241 (29%)	2,044 (21%)	18,777 (32%)
18,000 to 30,999	103,456 (22%)	61,219 (21%)	10,268 (21%)	2,149 (22%)	10,580 (21%)	2,653 (24%)	2,136 (22%)	14,451 (25%)
31,000 to 51,999	107,619 (23%)	69,485 (24%)	10,951 (23%)	1,858 (19%)	12,113 (25%)	2,034 (18%)	2,226 (23%)	8,952 (15%)
52,000 to 100,000	84,396 (18%)	57,717 (20%)	8,967 (19%)	1,295 (13%)	9,353 (19%)	1,186 (11%)	1,724 (17%)	4,154 (7.2%)
Greater than 100,000	22,458 (4.7%)	16,513 (5.7%)	2,244 (4.7%)	290 (3.0%)	2,203 (4.5%)	220 (2.0%)	350 (3.5%)	638 (1.1%)
Employment status: N (%)								
Unemployed	39,015 (8.2%)	21,425 (7.4%)	4,518 (9.4%)	1,498 (15%)	4,460 (9.0%)	1,488 (13%)	1,126 (11%)	4,500 (7.7%)
Eemployed	278,026 (58%)	178,312 (62%)	28,522 (59%)	5,137 (53%)	32,987 (67%)	5,500 (49%)	6,165 (62%)	21,403 (37%)
Retired	155,193 (33%)	87,378 (30%)	14,803 (31%)	2,964 (30%)	11,451 (23%)	4,086 (36%)	2,451 (25%)	32,060 (55%)
Sleep duration (h/day)	7.10 (1.28)	7.13 (1.21)	7.03 (1.33)	6.93 (1.56)	7.05 (1.30)	7.03 (1.49)	7.00 (1.40)	7.09 (1.44)
Smoking: N (%)								
Never	263,799 (55%)	170,126 (59%)	27,828 (58%)	4,828 (49%)	27,930 (57%)	3,741 (33%)	5,374 (54%)	23,972 (41%)
Previous	161,821 (34%)	91,654 (32%)	17,018 (35%)	3,312 (34%)	17,765 (36%)	4,460 (40%)	3,050 (31%)	24,562 (42%)
Current	49,368 (10%)	27,068 (9.3%)	3,233 (6.7%)	1,592 (16%)	3,526 (7.1%)	2,987 (27%)	1,427 (14%)	9,535 (16%)
Drinking: N (%)								
Never	21,025 (4.4%)	11,521 (4.0%)	2,132 (4.4%)	704 (7.2%)	1,958 (4.0%)	713 (6.3%)	716 (7.2%)	3,281 (5.6%)
Previous	16,508 (3.5%)	8,557 (3.0%)	1,967 (4.1%)	563 (5.8%)	1,496 (3.0%)	542 (4.8%)	424 (4.3%)	2,959 (5.1%)
Current	438,640 (92%)	269,384 (93%)	44,095 (91%)	8,483 (87%)	45,872 (93%)	9,958 (89%)	8,740 (88%)	52,108 (89%)
IPAQ_activity: N (%)								
low	72,171 (19%)	42,571 (18%)	7,814 (20%)	1,740 (23%)	8,501 (21%)	1,767 (20%)	1,725 (22%)	8,053 (18%)
moderate	157,544 (41%)	97,314 (41%)	15,969 (41%)	3,180 (41%)	16,662 (42%)	3,487 (40%)	3,103 (39%)	17,829 (40%)
high	156,159 (40%)	97,687 (41%)	15,359 (39%)	2,800 (36%)	14,879 (37%)	3,518 (40%)	3,105 (39%)	18,811 (42%)
Vascularheart problems: N (%)								
Angina	10,355 (2.2%)	4,793 (1.7%)	1,139 (2.4%)	325 (3.3%)	911 (1.8%)	358 (3.2%)	226 (2.3%)	2,603 (4.5%)
High blood pressure	118,366 (25%)	66,959 (23%)	11,518 (24%)	2,589 (27%)	12,559 (25%)	3,194 (28%)	2,386 (24%)	19,161 (33%)
Cancer	103,109 (22%)	61,214 (21%)	9,945 (21%)	1,987 (20%)	9,315 (19%)	2,606 (23%)	1,929 (20%)	16,113 (28%)
Diabetes	22,842 (4.8%)	11,868 (4.1%)	2,023 (4.2%)	681 (7.0%)	2,283 (4.6%)	930 (8.3%)	547 (5.5%)	4,510 (7.7%)
Asthma	50,863 (11%)	30,359 (10%)	5,677 (12%)	1,134 (12%)	5,707 (12%)	1,034 (9.2%)	1,230 (12%)	5,722 (9.8%)
Hayfever, allergic rhinitis or eczema	83,705 (18%)	51,941 (18%)	9,839 (20%)	1,833 (19%)	9,498 (19%)	1,538 (14%)	1,822 (18%)	7,234 (12%)
Medication: N (%)								
Cholesterol lowering medication	30,562 (12%)	15,412 (9.8%)	3,373 (12%)	851 (14%)	3,098 (10%)	889 (16%)	537 (11%)	6,402 (21%)
Blood pressure medication	27,244 (10%)	15,095 (9.6%)	2,855 (9.9%)	661 (11%)	3,086 (10%)	609 (11%)	494 (10%)	4,444 (15%)
Insulin and Oral contraceptive pill or minipill	6,528 (2.5%)	4,345 (2.8%)	782 (2.7%)	113 (1.8%)	921 (3.0%)	76 (1.4%)	135 (2.7%)	156 (0.5%)
Hormone replacement therapy	15,207 (5.8%)	9,302 (5.9%)	1,945 (6.7%)	360 (5.8%)	1,635 (5.3%)	319 (5.8%)	308 (6.2%)	1,338 (4.4%)
Pain relief medication	203,858 (43%)	112,555 (39%)	24,915 (52%)	5,330 (55%)	22,085 (45%)	5,172 (46%)	5,240 (53%)	28,561 (49%)
BMI (kg/m^2^)	27.36 (4.78)	27.12 (4.64)	27.20 (4.89)	27.69 (5.23)	27.80 (5.02)	28.04 (5.18)	27.70 (5.10)	28.10 (4.81)
WHR	0.87 (0.09)	0.87 (0.09)	0.86 (0.09)	0.87 (0.09)	0.86 (0.09)	0.89 (0.09)	0.88 (0.09)	0.89 (0.09)
DBP (mmHg)	82.31 (10.68)	82.36 (10.68)	81.44 (10.58)	81.36 (10.75)	82.46 (10.74)	82.76 (10.95)	82.13 (10.79)	82.75 (10.57)
SBP (mmHg)	139.75 (19.68)	139.47 (19.60)	137.65 (19.22)	137.58 (19.58)	138.00 (19.40)	141.68 (20.06)	137.68 (19.34)	144.83 (19.88)
Cholesterol (mmol/L)	5.74 (1.13)	5.75 (1.11)	5.74 (1.12)	5.69 (1.15)	5.73 (1.11)	5.70 (1.18)	5.66 (1.13)	5.70 (1.19)
CRP (mg/L)	2.57 (4.32)	2.39 (4.11)	2.65 (4.45)	3.01 (4.93)	2.60 (4.11)	3.29 (5.17)	2.74 (4.59)	3.18 (4.91)
Glucose (mmol/L)	5.11 (1.20)	5.08 (1.15)	5.08 (1.16)	5.15 (1.37)	5.09 (1.24)	5.25 (1.55)	5.11 (1.34)	5.24 (1.33)
HDL-C (mmol/L)	1.46 (0.38)	1.47 (0.38)	1.46 (0.38)	1.44 (0.38)	1.46 (0.38)	1.40 (0.38)	1.39 (0.37)	1.41 (0.37)
Direct LDL (mmol/L)	3.59 (0.86)	3.59 (0.85)	3.59 (0.85)	3.56 (0.87)	3.58 (0.85)	3.58 (0.90)	3.56 (0.86)	3.57 (0.90)
Triglycerides (mmol/L)	1.74 (1.02)	1.71 (1.01)	1.75 (1.03)	1.76 (1.03)	1.73 (1.04)	1.87 (1.09)	1.81 (1.10)	1.88 (1.03)

mean ± standard deviation for continuous variables and frequency and percentage for categorical variables

### Association between different types of oral conditions with stroke, IS and MI

**Fig. 2:**
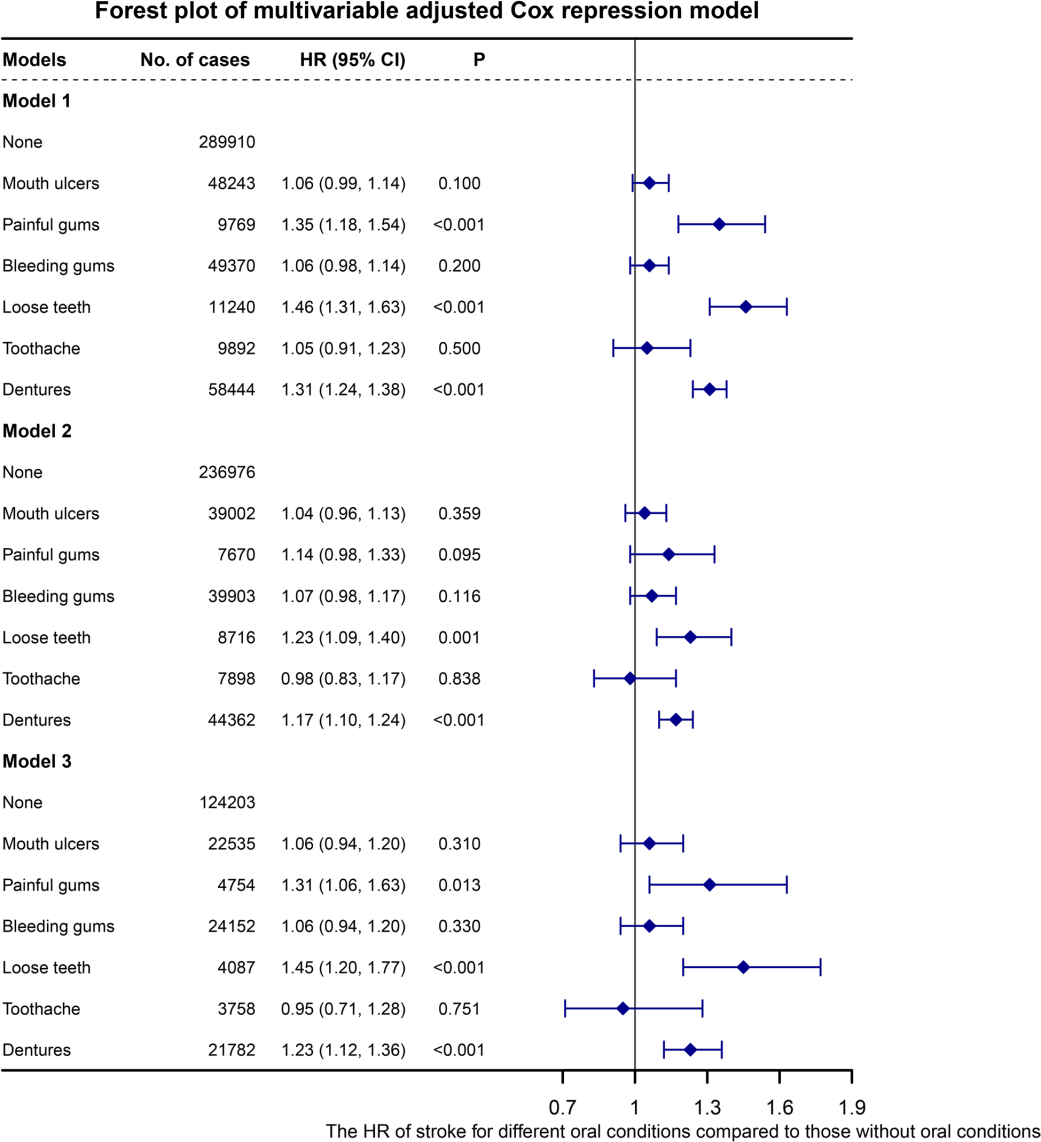
The association between different types of oral conditions and stroke incidence for three multivariable models. Notes: Sample sizes vary across models due to complete-case analysis

The associations of different types of oral conditions with stroke risk are shown in Table 2. In the minimally adjusted model, participants with specific oral conditions showed an elevated stroke risk compared to those with no oral conditions. Notably, conditions indicative of periodontal pathology demonstrated the strongest associations, participants with painful gums [HR_painful gums_: 1.35 (1.18, 1.54)] and loose teeth [HR_loose teeth_: 1.46 (1.31, 1.63)] [HR_dentures_: 1.31 (1.24, 1.38)] have higher stroke risk. Additionally, dentures as a marker of end-stage tooth loss were associated with increased stroke risk and dentures (Fig. 2). After additionally adjusted for lifestyle factors and Townsend deprivation index, employment status and household income, the associations between loose teeth [HR_loose teeth_: 1.23 (1.09, 1.40)] and dentures [HR_dentures_: 1.17 (1.10, 1.24)] with stroke risk were attenuated but still remained significant (Fig. 2). In the fully adjusted model (Model 3), we observed consistent patterns across the conceptual categories: periodontal conditions (painful gums [HR_painful gums_: 1.31 (1.06, 1.63)], loose teeth [HR_loose teeth_: 1.45 (1.20, 1.77)]) and end-stage tooth loss (dentures [HR_dentures_: 1.23 (1.12, 1.36)]) maintained significant associations with stroke risk (Fig. 2). In contrast, transient conditions (mouth ulcers and toothache) and other periodontal indicators (bleeding gums) showed no significant associations in any model. After FDR correction for multiple testing, loose teeth and dentures (both *P*_FDR_Adjusted_P_Value_ < 0.001) and painful gums (*P*_FDR_Adjusted_P_Value_ = 0.026) remained significantly associated with stroke risk (Table 2). When examining IS specifically, the pattern of associations for the more advanced oral conditions persisted. Both periodontal disease indicators loose teeth [HR_loose teeth_: 1.53 (1.23, 1.91)] and end-stage tooth loss dentures [HR_dentures_: 1.25 (1.12, 1.40)] remained significantly associated with IS risk after full adjustment (Supplementary Table 4). For myocardial infarction (MI) incidence, a distinct pattern emerged. Transient conditions (toothache) and one periodontal indicator (bleeding gums) were not associated with MI risk. However, the other four oral conditions—encompassing both periodontal indicators (painful gums, loose teeth) and end-stage tooth loss (dentures), as well as the transient condition of mouth ulcers—were significantly associated with MI (Supplementary Table 5).

**Table 2:** Association between different types of oral conditions with stroke risk

Mouth oral conditions	Model 1		Model 2		Model 3		
	**HR 95*CI*%**	***P***	**HR 95*CI*%**	***P***	**HR 95*CI*%**	***P***	***P_FDR***
None	1.00	Ref	1	Ref	1.00	Ref	
Mouth ulcers	1.06 (0.99, 1.14)	0.100	1.04 (0.96, 1.13)	0.359	1.06 (0.94, 1.20)	0.310	0.396
Painful gums	1.35 (1.18, 1.54)	< 0.001	1.14 (0.98, 1.33)	0.095	1.31 (1.06, 1.63)	0.013	0.026*
Bleeding gums	1.06 (0.98, 1.14)	0.200	1.07 (0.98, 1.17)	0.116	1.06 (0.94, 1.20)	0.330	0.396
Loose teeth	1.46 (1.31, 1.63)	< 0.001	1.23 (1.09, 1.40)	0.001	1.45 (1.20, 1.77)	< 0.001	< 0.001*
Toothache	1.05 (0.91, 1.23)	0.500	0.98 (0.83, 1.17)	0.838	0.95 (0.71, 1.28)	0.751	0.751
Dentures	1.31 (1.24, 1.38)	< 0.001	1.17 (1.10, 1.24)	< 0.001	1.23 (1.12, 1.36)	< 0.001	< 0.001*

^*^indicates associations that remained statistically significant after FDR adjustment for multiple testing

Sensitivity analyses results from complete-case analysis and multiple imputation produced consistent effect estimates and conclusions (Supplementary Table 6), supporting the reliability of our primary analysis. After excluding those with dentures, the key associations between other oral health indicators (e.g., mobile teeth, gum bleeding) and stroke risk remained statistically significant with consistent effect sizes (Supplementary Table 7).

Considering all the oral problems together, the PAR% for total stroke, IS and MI were 5.25%, 6.17% and 7.73%, respectively. These findings suggest that, assuming a causal relationship, improving oral health to eliminate the specific problems measured in this study could potentially lead to 5.25%, 6.17% and 7.73% reduction in the total number of total strokes, IS and MI cases. This highlights the significant potential population-level impact of improving oral health on reducing the burden of major cardiovascular diseases.

## Discussion

In this large prospective study, we observed that specific oral conditions were associated with an increased risk of stroke, IS, and MI. Conditions indicative of active periodontal disease (painful gums, loose teeth) and end-stage tooth loss (dentures) showed consistent positive associations, whereas transient conditions (mouth ulcers, toothache) generally showed null associations. Notably, the association was strongest for loose teeth, a marker of advanced periodontal disease. Individuals with loose teeth had a 46% higher risk of stroke in the baseline model. This hazard ratio attenuated only slightly to 45% after full adjustment for socioeconomic factors, indicating that while socioeconomic status explains a small portion of the association, the majority of the increased risk appears to be independent of these factors.

To the best of our knowledge, this was the first study to assess the association between different oral conditions and stroke, IS and MI. Previous epidemiological studies provided robust evidence between periodontitis and cardiovascular incidence and mortality, studies reported that compared to individuals without periodontitis or less severe periodontitis, periodontitis patients have a higher risk for first coronary event, stroke, heart failure, atrial fibrillation and cardiovascular mortality [11, 13, 18–20]. This study comprehensively explored the association between different oral conditions and events, helping to prioritize the high-risk groups for medical staff. Those without any oral conditions should keep healthy lifestyle, visit hospital annually for physical examination or dental clinic for dental care to prevent the occurrence of different oral conditions. However, for those with painful gums, loose teeth and dentures, they should pay attention to establish a healthy lifestyle, constantly visit the dental and medical practices, to actively seek for treatment and then lower their risk for stroke, MI and IS incidence. In addition, this study has significant implications in public health and disease prevention. This PAR% of stroke was 5.25%, which suggests that if oral conditions were causally related to stroke incidence and if these conditions could be entirely eliminated from the population, the theoretical maximum reduction in stroke incidence would be 5.25%, not an intervention target. This highlights the potential population-level importance of preventing oral conditions, though this hypothetical scenario does not imply a proven causal relationship.

The underlying mechanisms between oral conditions and stroke are currently unclear. However, as a multifactorial chronic systemic inflammatory disease, periodontitis was proved to be associated with elevated levels of a series of systemic inflammation markers, such as interleukin-6, CRP and TNF alpha [21, 22]. Evidence further suggests that the elevated inflammatory factors in periodontitis patients may increase inflammatory activity in atherosclerotic lesions, potentially increasing the risk for systemic and neurological diseases, such as cardiovascular events [23], rheumatoid arthritis [24], and Alzheimer’s disease [25]. Periodontitis, a chronic inflammatory disease of the supporting tissues of the teeth, is a primary underlying cause of many of the conditions we investigated. In large-scale epidemiological studies where clinical periodontal examinations are not feasible, indicators such as tooth loss and tooth mobility are widely used as surrogate markers for severe periodontitis [26, 27]. This is justified because periodontitis is the leading cause of tooth loss in adults [27], and self-reports of these conditions have been validated against clinical diagnoses [28]. Therefore, in the present study, these self-reported oral conditions are interpreted as reflecting the cumulative burden of chronic periodontal disease. For instance, loose teeth are a direct symptom of advanced periodontitis, resulting from the destruction of the periodontal ligament and alveolar bone. Similarly, painful or bleeding gums are classic signs of gingivitis, the reversible precursor to periodontitis [29]. Although not all gingivitis progresses to periodontitis, its presence indicates a state of oral inflammation. Furthermore, dentures are often the ultimate consequence of severe periodontitis (and caries) leading to tooth loss [30]. Therefore, the associations we observed, particularly for loose teeth and dentures, likely capture the long-term systemic inflammatory burden and bacterial load associated with chronic periodontitis [31]. This provides a plausible biological pathway linking our findings to an increased risk of stroke.

The lack of significant association for transient conditions like toothache and bleeding gums can be explained by their nature. These are often acute or intermittent symptoms that may not reflect the same level of sustained, systemic biological insult as permanent conditions like tooth loss (represented by dentures) or mobile teeth. While bleeding gums can be a sign of gingivitis (a reversible inflammation), its weaker association suggests that the stroke risk may be more strongly linked to the chronic, tissue-destructive processes of periodontitis, of which tooth mobility is a key clinical sign [32]. The transient nature of pain and bleeding means they are less likely to capture the long-term inflammatory burden hypothesized to drive cardiovascular risk [33]. Beyond direct biological pathways, the observed associations may also reflect cumulative life-course disadvantages. Socioeconomic disparities and early-life adversity can drive both oral health decline and systemic inflammation over time. Oral symptoms thus serve as a "sentinel marker" of long-term inflammatory burden, where social determinants and chronic stress converge to amplify cardiovascular risk.

Notably, dentures represent a treatment state rather than an active disease. While they may indicate historical disease severity, their presence also reflects healthcare access and treatment-seeking behavior. This dual role necessitates distinguishing between etiology and consequence. In our main analysis, dentures are interpreted as a proxy for cumulative disease burden, supported by sensitivity analyses excluding denture users that showed consistent associations with stroke risk.

Important strength should be mentioned for the current study. This is the first large cohort study to estimate the association between different types of oral conditions and stroke, IS and MI incidence, this association provides novel insights into future disease prevention and offers an opportunity for researchers to investigate the relationship between oral conditions and other chronic disease. Our study has several limitations that should be considered when interpreting the results. First, the assessment of oral health relied on self-reported data without clinical validation. These self-reported oral conditions reflect symptom experiences rather than clinically diagnosed diseases; severity, chronicity, and treatment history remain unmeasured. This approach is susceptible to recall bias and non-differential misclassification, as participants may not accurately remember or report their symptoms. Additionally, differential reporting by socioeconomic status could introduce residual confounding, though non-differential misclassification remains the primary bias toward the null. This limitation likely attenuates the observed effect estimates toward the null, meaning our reported associations are likely conservative. Second, dental status was only assessed at baseline. This single-time-point measurement does not account for changes in oral health over the follow-up period, which could introduce exposure misclassification and potentially attenuate the observed associations toward the null. Future studied should involve the investigation of the cumulative effect of multiple oral health conditions with stroke risk. Third, despite adjusting for a range of important covariates, we cannot rule out residual confounding by unmeasured or measured factors. For instance, aspects of diet, oral hygiene practices, or access to dental care that were not fully captured in the data could influence our results. Finally, the UK Biobank’s healthy volunteer bias likely underestimates absolute stroke risk and true effect sizes. Given the strong socioeconomic gradient in oral disease, associations may be more pronounced in disadvantaged populations—where oral pathology burden and limited care access converge—highlighting reduced generalizability to lower-income or non-Western groups. Despite these limitations, the large sample size, prospective design, and detailed data on numerous confounders are key strengths of our study. The observed associations provide a valuable foundation for future research incorporating repeated clinical dental assessments to better establish temporality and causality. Notably, negative control exposures showed no significant association with stroke risk in the fully adjusted model, enhancing the specificity and plausibility of the observed link between oral conditions and stroke.

## Conclusions

In this large prospective study, we found that advanced oral conditions—particularly markers of periodontal disease (loose teeth) and end-stage tooth loss (dentures)—were significantly associated with increased risks of stroke and myocardial infarction. These associations may reflect oral health status as a correlate of broader health vulnerability, including cumulative social and behavioral factors. While oral conditions could serve as a practical indicator for identifying individuals at higher cardiovascular risk. Screening for these oral conditions could help prioritize high-risk populations for more targeted cardiovascular prevention strategies, though residual confounding by social factors cannot be fully excluded.

## Supplementary Information

Supplementary Material 1.

## Data Availability

Data are available in a public, open access repository. All data used in this study were accessed from the publicly available UK Biobank Resource under application number 71051. These data cannot be shared with other investigators.
