# Supplementary material for: Associations between self-reported oral health and incident stroke: a prospective analysis of the UK Biobank
Source: BMC Public Health. 2026 Feb 5;26:831. doi: 10.1186/s12889-026-26397-2 (PMC12973887; doi:10.1186/s12889-026-26397-2)
Supplement: Supplementary file 1 — Supplementary Material 1. [file 12889_2026_26397_MOESM1_ESM.docx]

**Table S1 Baseline characteristics of Study Patients with Oral Conditions by Stroke Status**

| **Characteristic** | **Overall**, N = 186,958*^1^* | Healthy, N = 182,543*^1^* | **Stroke**, N = 4,415*^1^* | ***P*-value*** |
| --- | --- | --- | --- | --- |
| Age (years) | 57.27 (8.07) | 57.16 (8.07) | 61.68 (6.60) | <0.001 |
| Female: N (%) | 106,834 (57%) | 104,721 (57%) | 2,113 (48%) | <0.001 |
| White: N (%) | 174,627 (93%) | 170,471 (93%) | 4,156 (94%) | <0.001 |
| Education score | 17.36 (17.16) | 17.30 (17.12) | 20.13 (18.85) | <0.001 |
| Townsend deprivation index | -0.99 (3.22) | -1.00 (3.22) | -0.64 (3.38) | <0.001 |
| Household income: N (%) ($) |  |  |  | <0.001 |
| Less than 18,000 | 44,565 (24%) | 43,043 (24%) | 1,522 (35%) |  |
| 18,000 to 30,999 | 42,237 (23%) | 41,215 (23%) | 1,022 (23%) |  |
| 31,000 to 51,999 | 38,134 (20%) | 37,465 (21%) | 669 (15%) |  |
| 52,000 to 100,000 | 26,679 (14%) | 26,361 (14%) | 318 (7.3%) |  |
| Greater than 100,000 | 5,945 (3.2%) | 5,875 (3.2%) | 70 (1.6%) |  |
| Employment status: N (%) |  |  |  | <0.001 |
| Unemployed | 17,590 (9.4%) | 17,146 (9.4%) | 444 (10%) |  |
| In paid employment or self-employed | 99,714 (53%) | 98,207 (54%) | 1,507 (34%) |  |
| Retired | 67,815 (36%) | 65,385 (36%) | 2,430 (55%) |  |
| Smoking: N (%) |  |  |  | <0.001 |
| Never | 93,673 (50%) | 91,884 (50%) | 1,789 (41%) |  |
| Previous | 70,167 (38%) | 68,384 (37%) | 1,783 (40%) |  |
| Current | 22,300 (12%) | 21,492 (12%) | 808 (18%) |  |
| IPAQ_activity: N (%) |  |  |  | 0.016 |
| low | 29,600 (20%) | 28,859 (20%) | 741 (22%) |  |
| moderate | 60,230 (41%) | 58,907 (41%) | 1,323 (39%) |  |
| high | 58,472 (39%) | 57,131 (39%) | 1,341 (39%) |  |
| Drinking: N (%) |  |  |  | <0.001 |
| Never | 9,504 (5.1%) | 9,231 (5.1%) | 273 (6.2%) |  |
| Previous | 7,951 (4.3%) | 7,682 (4.2%) | 269 (6.1%) |  |
| Current | 169,256 (91%) | 165,388 (91%) | 3,868 (88%) |  |
| Multimorbidity: N (%) |  |  |  | <0.001 |
| Cancer | 41,895 (22%) | 40,516 (22%) | 1,379 (31%) |  |
| Diabetes: | 10,974 (5.9%) | 10,413 (5.7%) | 561 (13%) |  |
| Angina | 5,562 (3.0%) | 5,281 (2.9%) | 281 (6.4%) |  |
| High blood pressure | 51,407 (27%) | 49,637 (27%) | 1,770 (40%) |  |
| Asthma | 20,504 (11%) | 20,065 (11%) | 439 (9.9%) |  |
| Hayfever, allergic rhinitis or eczema | 31,764 (17%) | 31,167 (17%) | 597 (14%) |  |
| Medication : N (%) |  |  |  | <0.001 |
| Cholesterol lowering medication | 15,150 (14%) | 14,625 (14%) | 525 (25%) |  |
| Blood pressure medication | 12,149 (11%) | 11,791 (11%) | 358 (17%) |  |
| Insulin and Oral contraceptive pill or minipill | 2,183 (2.0%) | 2,166 (2.1%) | 17 (0.8%) |  |
| Hormone replacement therapy | 5,905 (5.5%) | 5,812 (5.6%) | 93 (4.4%) |  |
| Pain relief medication | 91,303 (49%) | 88,836 (49%) | 2,467 (56%) |  |
| WHR | 0.87 (0.09) | 0.87 (0.09) | 0.90 (0.09) | <0.001 |
| Sleep duration | 7.05 (1.38) | 7.05 (1.38) | 7.07 (1.57) | 0.041 |
| DBP | 82.22 (10.68) | 82.18 (10.65) | 84.15 (11.45) | <0.001 |
| SBP | 140.18 (19.81) | 140.00 (19.74) | 147.79 (21.30) | <0.001 |
| BMI (kg/m^2^) | 27.74 (4.96) | 27.72 (4.95) | 28.42 (5.22) | <0.001 |
| Cholesterol (mmol/L) | 5.72 (1.15) | 5.72 (1.15) | 5.58 (1.19) | <0.001 |
| CRP (mg/L) | 2.87 (4.60) | 2.85 (4.60) | 3.42 (4.76) | <0.001 |
| Glucose (mmol/L) | 5.15 (1.28) | 5.14 (1.26) | 5.45 (1.90) | <0.001 |
| HDL-C (mmol/L) | 1.44 (0.38) | 1.44 (0.38) | 1.37 (0.38) | <0.001<0.001 |
| Direct LDL (mmol/L) | 3.58 (0.87) | 3.58 (0.87) | 3.50 (0.90) | <0.001 |
| Triglycerides (mmol/L) | 1.79 (1.04) | 1.79 (1.04) | 1.91 (1.06) | <0.001 |

mean ± standard deviation for continuous variables and frequency and percentage for categorical variables.

* Pearson’s Chi-squared test; Wilcoxon rank sum test

**Table S2 Baseline characteristics of Study Patients with Oral Conditions by Ischemic Stroke Status**

| **Characteristic** | **Overall**, N = 186,958 | **Healthy individuals**, N = 183,388 | **Ischemic Stroke**, N = 3,570 | ***P*-value*** |
| --- | --- | --- | --- | --- |
| Age (years) | 57.27 (8.07) | 57.18 (8.07) | 61.97 (6.42) | <0.001 |
| White: N (%) | 174,627 (93%) | 171,266 (93%) | 3,361 (94%) | <0.001 |
| Female: N (%) | 106,834 (57%) | 105,228 (57%) | 1,606 (45%) | <0.001 |
| Townsend deprivation index | -0.99 (3.22) | -0.99 (3.22) | -0.60 (3.41) | <0.001 |
| Education score | 17.36 (17.16) | 17.30 (17.12) | 20.46 (19.06) | <0.001 |
| Household income: N (%) ($) |  |  |  | <0.001 |
| Less than 18,000 | 44,565 (24%) | 43,303 (24%) | 1,262 (36%) |  |
| 18,000 to 30,999 | 42,237 (23%) | 41,401 (23%) | 836 (24%) |  |
| 31,000 to 51,999 | 38,134 (20%) | 37,604 (21%) | 530 (15%) |  |
| 52,000 to 100,000 | 26,679 (14%) | 26,448 (14%) | 231 (6.5%) |  |
| Greater than 100,000 | 5,945 (3.2%) | 5,893 (3.2%) | 52 (1.5%) |  |
| Employment status: N (%) |  |  |  | <0.001 |
| Unemployed | 17,590 (9.4%) | 17,226 (9.4%) | 364 (10%) |  |
| In paid employment or self-employed | 99,714 (53%) | 98,545 (54%) | 1,169 (33%) |  |
| Retired | 67,815 (36%) | 65,807 (36%) | 2,008 (56%) |  |
| Smoking: N (%) |  |  |  | <0.001 |
| Never | 93,673 (50%) | 92,241 (50%) | 1,432 (40%) |  |
| Previous | 70,167 (38%) | 68,720 (37%) | 1,447 (41%) |  |
| Current | 22,300 (12%) | 21,634 (12%) | 666 (19%) |  |
| Drinking: N (%) |  |  |  | <0.001 |
| Never | 9,504 (5.1%) | 9,287 (5.1%) | 217 (6.1%) |  |
| Previous | 7,951 (4.3%) | 7,728 (4.2%) | 223 (6.2%) |  |
| Current | 169,256 (91%) | 166,130 (91%) | 3,126 (88%) |  |
| IPAQ_activity |  |  |  | 0.015 |
| low | 29,600 (20%) | 28,994 (20%) | 606 (22%) |  |
| moderate | 60,230 (41%) | 59,164 (41%) | 1,066 (39%) |  |
| high | 58,472 (39%) | 57,384 (39%) | 1,088 (39%) |  |
| Multimorbidity: N (%) |  |  |  | <0.001 |
| Diabetes | 10,974 (5.9%) | 10,470 (5.7%) | 504 (14%) |  |
| Angina | 5,562 (3.0%) | 5,315 (2.9%) | 247 (6.9%) |  |
| High blood pressure | 51,407 (27%) | 49,935 (27%) | 1,472 (41%) |  |
| Cancer | 41,895 (22%) | 40,777 (22%) | 1,118 (31%) |  |
| Asthma | 20,504 (11%) | 20,143 (11%) | 361 (10%) |  |
| Hayfever, allergic rhinitis or eczema | 31,764 (17%) | 31,301 (17%) | 463 (13%) |  |
| Medication: N (%) |  |  |  | <0.001 |
| Cholesterol lowering medication | 15,150 (14%) | 14,712 (14%) | 438 (27%) |  |
| Blood pressure medication | 12,149 (11%) | 11,864 (11%) | 285 (18%) |  |
| Insulin and Oral contraceptive pill or minipill | 2,183 (2.0%) | 2,176 (2.1%) | 7 (0.4%) |  |
| Hormone replacement therapy | 5,905 (5.5%) | 5,832 (5.5%) | 73 (4.5%) |  |
| Pain relief medication | 91,303 (49%) | 89,293 (49%) | 2,010 (56%) |  |
| Waist_to_hip_ratio | 0.87 (0.09) | 0.87 (0.09) | 0.91 (0.09) | <0.001 |
| Sleep duration (h/day) | 7.05 (1.38) | 7.05 (1.38) | 7.08 (1.62) | <0.001 |
| DBP (mmHg) | 82.22 (10.68) | 82.18 (10.66) | 84.39 (11.41) | <0.001 |
| SBP (mmHg) | 140.18 (19.81) | 140.02 (19.75) | 148.48 (21.14) | <0.001 |
| BMI | 27.74 (4.96) | 27.72 (4.95) | 28.65 (5.26) | <0.001 |
| Cholesterol (mmol/L) | 5.72 (1.15) | 5.72 (1.15) | 5.56 (1.20) | <0.001 |
| CRP (mg/L) | 2.87 (4.60) | 2.85 (4.59) | 3.52 (4.91) | <0.001 |
| Glucose (mmol/L) | 5.15 (1.28) | 5.14 (1.26) | 5.50 (2.01) | <0.001 |
| HDL-C (mmol/L) | 1.44 (0.38) | 1.44 (0.38) | 1.35 (0.37) | <0.001 |
| Direct LDL (mmol/L) | 3.58 (0.87) | 3.58 (0.87) | 3.49 (0.91) | <0.001 |
| Triglycerides (mmol/L) | 1.79 (1.04) | 1.79 (1.04) | 1.95 (1.07) | <0.001 |

mean ± standard deviation for continuous variables and frequency and percentage for categorical variables.

* Pearson’s Chi-squared test; Wilcoxon rank sum test

**Table S3 Baseline characteristics of Study Patients with Oral Conditions by Myocardial Infarction Status**

| **Characteristic** | **Overall**, N = 186,958 | **Healthy Individuals**, N = 179,999 | **MI**, N = 6,959 | ***P*-value*** |
| --- | --- | --- | --- | --- |
| Age (years) | 57.27 (8.07) | 57.13 (8.08) | 60.82 (6.86) | <0.001 |
| Female: N (%) | 106,834 (57%) | 104,319 (58%) | 2,515 (36%) | <0.001 |
| White: N (%) | 174,627 (93%) | 168,128 (93%) | 6,499 (93%) | <0.001 |
| Townsend deprivation index | -0.99 (3.22) | -1.00 (3.21) | -0.54 (3.39) | <0.001 |
| Education score | 17.36 (17.16) | 17.23 (17.08) | 20.88 (18.87) | <0.001 |
| Household income: N (%) ($) |  |  |  | <0.001 |
| Less than 18,000 | 44,565 (24%) | 42,201 (24%) | 2,364 (34%) |  |
| 18,000 to 30,999 | 42,237 (23%) | 40,665 (23%) | 1,572 (23%) |  |
| 31,000 to 51,999 | 38,134 (20%) | 37,006 (21%) | 1,128 (16%) |  |
| 52,000 to 100,000 | 26,679 (14%) | 26,104 (15%) | 575 (8.3%) |  |
| Greater than 100,000 | 5,945 (3.2%) | 5,849 (3.3%) | 96 (1.4%) |  |
| Employment status: N (%) |  |  |  | <0.001 |
| Unemployed | 17,590 (9.4%) | 16,763 (9.3%) | 827 (12%) |  |
| In paid employment or self-employed | 99,714 (53%) | 97,044 (54%) | 2,670 (38%) |  |
| Retired | 67,815 (36%) | 64,415 (36%) | 3,400 (49%) |  |
| Smoking: N (%) |  |  |  | <0.001 |
| Never | 93,673 (50%) | 91,088 (51%) | 2,585 (37%) |  |
| Previous | 70,167 (38%) | 67,221 (37%) | 2,946 (42%) |  |
| Current | 22,300 (12%) | 20,911 (12%) | 1,389 (20%) |  |
| Drinking: N (%) |  |  |  | <0.001 |
| Never | 9,504 (5.1%) | 9,078 (5.0%) | 426 (6.1%) |  |
| Previous | 7,951 (4.3%) | 7,489 (4.2%) | 462 (6.6%) |  |
| Current | 169,256 (91%) | 163,201 (91%) | 6,055 (87%) |  |
| IPAQ_activity |  |  |  | <0.001 |
| low | 29,600 (20%) | 28,345 (20%) | 1,255 (23%) |  |
| moderate | 60,230 (41%) | 58,137 (41%) | 2,093 (39%) |  |
| high | 58,472 (39%) | 56,413 (39%) | 2,059 (38%) |  |
| Multimorbidity: N (%) |  |  |  |  |
| Diabetes | 10,974 (5.9%) | 9,962 (5.5%) | 1,012 (15%) |  |
| Angina | 5,562 (3.0%) | 4,622 (2.6%) | 940 (14%) |  |
| High blood pressure | 51,407 (27%) | 48,845 (27%) | 2,562 (37%) |  |
| Cancer | 41,895 (22%) | 39,916 (22%) | 1,979 (28%) |  |
| Asthma | 20,504 (11%) | 19,690 (11%) | 814 (12%) |  |
| Hayfever, allergic rhinitis or eczema | 31,764 (17%) | 30,876 (17%) | 888 (13%) |  |
| Medication: N (%) |  |  |  | <0.001 |
| Cholesterol lowering medication | 15,150 (14%) | 14,359 (14%) | 791 (31%) |  |
| Blood pressure medication | 12,149 (11%) | 11,718 (11%) | 431 (17%) |  |
| Insulin and Oral contraceptive pill or minipill | 2,183 (2.0%) | 2,159 (2.1%) | 24 (1.0%) |  |
| Hormone replacement therapy | 5,905 (5.5%) | 5,791 (5.6%) | 114 (4.5%) |  |
| Pain relief medication | 91,303 (49%) | 87,108 (48%) | 4,195 (60%) |  |
| Waist_to_hip_ratio | 0.87 (0.09) | 0.87 (0.09) | 0.92 (0.09) | <0.001 |
| Sleep duration (h/day) | 7.05 (1.38) | 7.05 (1.37) | 7.04 (1.61) | >0.9 |
| DBP (mmHg) | 82.22 (10.68) | 82.17 (10.65) | 83.66 (11.19) | <0.001 |
| SBP (mmHg) | 140.18 (19.81) | 139.93 (19.75) | 146.83 (20.36) | <0.001 |
| BMI | 27.74 (4.96) | 27.70 (4.95) | 28.83 (5.06) | <0.001 |
| Cholesterol (mmol/L) | 5.72 (1.15) | 5.72 (1.14) | 5.64 (1.29) | <0.001 |
| CRP (mg/L) | 2.87 (4.60) | 2.83 (4.55) | 3.73 (5.67) | <0.001 |
| Glucose (mmol/L) | 5.15 (1.28) | 5.13 (1.24) | 5.51 (2.06) | <0.001 |
| HDL-C (mmol/L) | 1.44 (0.38) | 1.44 (0.38) | 1.30 (0.35) | <0.001 |
| Direct LDL (mmol/L) | 3.58 (0.87) | 3.58 (0.87) | 3.58 (0.98) | 0.4 |
| Triglycerides (mmol/L) | 1.79 (1.04) | 1.78 (1.04) | 2.10 (1.14) | <0.001 |

mean ± standard deviation for continuous variables and frequency and percentage for categorical variables.

* Pearson’s Chi-squared test; Wilcoxon rank sum test

**Table S4 The association between different types of Oral Conditions and IS incidence for three multivariable models.**

| **Mouth dental problems** | **Model 1** | | **Model 2** | | **Model 3** | |
| --- | --- | --- | --- | --- | --- | --- |
|  | **HR 95*CI*%** | ***P*** | **HR 95*CI*%** | ***P*** | **HR 95*CI*%** | ***P*** |
| **None** | 1.00 | Ref | 1 | Ref | 1.00 | Ref |
| **Mouth ulcers** | 1.06 (0.98, 1.15) | <0.001 | 1.06 (0.96, 1.16) | 0.253 | 1.08 (0.94, 1.24) | 0.278 |
| **Painful gums** | 1.34 (1.16, 1.56) | <0.001 | 1.12 (0.94, 1.34) | 0.209 | 1.28 (1.00, 1.65) | 0.051 |
| **Bleeding gums** | 1.08 (0.99, 1.18) | 0.070 | 1.11 (1.00, 1.22) | 0.041 | 1.09 (0.94, 1.26) | 0.240 |
| **Loose teeth** | 1.53 (1.36, 1.72) | <0.001 | 1.28 (1.12, 1.47) | <0.001 | 1.53 (1.23, 1.91) | <0.001 |
| **Toothache** | 1.16 (0.98, 1.36) | 0.080 | 1.09 (0.91, 1.31) | 0.363 | 0.99 (0.70, 1.38) | 0.936 |
| **Dentures** | 1.36 (1.28, 1.44) | <0.001 | 1.21 (1.13, 1.29) | <0.001 | 1.25 (1.12, 1.40) | <0.001 |

**Table S5 The association between different types of Oral Conditions and MI incidence for three multivariable models.**

| **Mouth dental problems** | **Model 1** | | **Model 2** | | **Model 3** | |
| --- | --- | --- | --- | --- | --- | --- |
|  | **HR 95*CI*%** | ***P*** | **HR 95*CI*%** | ***P*** | **HR 95*CI*%** | ***P*** |
| **None** | 1.00 | Ref | 1 | Ref | 1.00 | Ref |
| **Mouth ulcers** | 1.14 (1.07, 1.20) | <0.001 | 1.12 (1.05, 1.19) | <0.001 | 1.26 (1.14, 1.40) | <0.001 |
| **Painful gums** | 1.63 (1.47, 1.80) | <0.001 | 1.39 (1.24, 1.56) | <0.001 | 1.50 (1.25, 1.81) | <0.001 |
| **Bleeding gums** | 1.05 (0.98, 1.11) | 0.152 | 1.05 (0.98, 1.12) | 0.183 | 1.12 (1.00, 1.26) | 0.053 |
| **Loose teeth** | 1.50 (1.37, 1.63) | <0.001 | 1.21 (1.10, 1.34) | <0.001 | 1.52 (1.28, 1.82) | <0.001 |
| **Toothache** | 1.19 (1.07, 1.33) | 0.002 | 1.09 (0.96, 1.24) | 0.178 | 1.02 (0.78, 1.33) | 0.897 |
| **Dentures** | 1.40 (1.34, 1.46) | <0.001 | 1.21 (1.15, 1.27) | <0.001 | 1.13 (1.03, 1.25) | 0.009 |

| **Mouth dental problems** | **Model 3** | | |
| --- | --- | --- | --- |
|  | ***N*** | **HR 95*CI*%** | ***P*** |
| **None** | 289910 | 1.00 | Ref |
| **Mouth ulcers** | 48243 | 1.03 (0.96, 1.10) | 0.467 |
| **Painful gums** | 9769 | 1.16 (1.01, 1.32) | 0.030 |
| **Bleeding gums** | 49370 | 1.04 (0.96, 1.12) | 0.361 |
| **Loose teeth** | 11240 | 1.20 (1.08, 1.34) | 0.001 |
| **Toothache** | 9892 | 0.95 (0.82, 1.11) | 0.534 |
| **Dentures** | 58444 | 1.13 (1.07, 1.19) | <0.001 |

**Table S6 The association between different types of Oral Conditions and stroke incidence for multivariable adjusted models using complete-case analysis.**

**Table S7 The association between different types of Oral Conditions (excluding dentures) and IS incidence for three multivariable models.**

| **Mouth dental problems** | **Model 1** | | **Model 2** | | **Model 3** | |
| --- | --- | --- | --- | --- | --- | --- |
|  | **HR 95*CI*%** | ***P*** | **HR 95*CI*%** | ***P*** | **HR 95*CI*%** | ***P*** |
| **None** | 1.00 | Ref | 1 | Ref | 1.00 | Ref |
| **Mouth ulcers** | 1.06 (0.99, 1.14) | 0.09 | 1.04 (0.96, 1.13) | 0.327 | 1.05 (0.93, 1.19) | 0.424 |
| **Painful gums** | 1.35 (1.18, 1.54) | <0.001 | 1.14 (0.98, 1.34) | 0.094 | 1.30 (1.05, 1.62) | 0.018 |
| **Bleeding gums** | 1.06 (0.98, 1.15) | 0.12 | 1.08 (0.99, 1.17) | 0.095 | 1.04 (0.92, 1.19) | 0.511 |
| **Loose teeth** | 1.45 (1.30, 1.62) | <0.001 | 1.22 (1.08, 1.39) | 0.002 | 1.43 (1.16, 1.75) | <0.001 |
| **Toothache** | 1.05 (0.91, 1.22) | 0.50 | 0.98 (0.82, 1.16) | 0.788 | 0.86 (0.63, 1.18) | 0.346 |
